# Moderating Effects of Endurance on Gait Speed Associations With Incident Mild Cognitive Impairment and Dementia

**DOI:** 10.1093/geroni/igaf122.1079

**Published:** 2025-12-31

**Authors:** Glen Myatt, Hunter Sylvester, Michael Griswold, Rebecca Gottesman, David Knopman, Kelley Pettee Gabriel, Kevin Sullivan, B Gwen Windham

**Affiliations:** University of Mississippi Medical Center, Jackson, Mississippi, United States; University of Mississippi Medical Center, Jackson, Mississippi, United States; University of Mississippi Medical Center - The MIND Center, Jackson, Mississippi, United States; National Institutes of Health, Bethesda, Maryland, United States; Mayo Clinic Department of Neurology, Rochester, Minnesota, United States; The University of Alabama at Birmingham, Birmingham, Alabama, United States; University of Mississippi Medical Center - The MIND Center, Jackson, Mississippi, United States; University of Mississippi Medical Center - The MIND Center, Jackson, Mississippi, United States

## Abstract

Although slower gait speed is associated with cognitive impairment, endurance may be more predictive in older adults with faster gait speed. We examined moderating effects of endurance on the relationship of gait speed with incident mild cognitive impairment (MCI) or dementia in cognitively normal ARIC participants (n = 2242, mean age=78.6 + 4.3, 58% female, 19% Black). At the sixth exam, endurance was assessed as distance walked at a fast pace (Two Minute Walk, 2MW); usual 4-meter gait speed was also assessed. Cognitive status was adjudicated during the 6th-9th exams (mean follow-up 5.1 years). Multinomial logistic regression models with a 2MW-x-gait speed interaction term estimated relative risk ratios (RRR) of MCI and dementia relative to normal cognition per SD higher gait speed or 2MW distance, adjusting for demographic and cardiovascular factors. Results used representative continuous gait speed and continuous 2MW distance values. Longer 2MW distance was associated with lower risk of MCI and dementia at gait speed <1m/s [e.g. gait speed=0.8: MCI RRR=0.59, 95%CI(0.42, 0.83); dementia RRR=0.29, (0.13, 0.63)] and with dementia at gait speed>1m/s [gait speed=1.2: RRR=0.28, (0.09, 0.81)], but not with MCI (RRR=0.85, (0.57, 1.26)]. Conversely, faster gait speed was associated with lower risk of MCI at low 2MW [100 m; RRR=0.84, (0.74, 0.94)], but was attenuated at high 2MW (150 m; RRR=0.91, (0.84, 1.00)]. A 2MW-x-gait speed interaction term was supported (p = 0.04), primarily driven by differential MCI associations. Combining endurance and gait speed may be especially helpful in identifying cognitively normal older adults at risk for impending early-stage cognitive impairment.

